# Maturation‐Associated Physiological and Biochemical Transitions in Carob (*Ceratonia siliqua* L.): Optimizing Harvest Timing Toward Functional Valorization of Fruits

**DOI:** 10.1155/tswj/5395647

**Published:** 2026-05-31

**Authors:** Salah Laaraj, Atman Adiba, Chaimae El-Rhouttais, Anas Hamdani, Wafae Sellami, Mohamed Kouighat, Abdelaziz Ed-Dra, Achraf Mamassi, Mohamed Lamsiah, Chaimaa Jabbari, Souad Salmaoui, Kaoutar Elfazazi

**Affiliations:** ^1^ Regional Center of Agricultural Research of Tadla, National Institute of Agricultural Research (INRA), Rabat, Morocco, inra.org.ma; ^2^ Environmental, Ecological, and Agro-Industrial Engineering Laboratory, LGEEAI, Sultan Moulay Slimane University (USMS), Faculty of Science and Technology (FST), Beni Mellal, Morocco, fst-usmba.ac.ma; ^3^ Laboratory Regional Center of Agricultural Research of Meknes, National Institute of Agricultural Research (INRA), Rabat, Morocco, inra.org.ma; ^4^ Laboratory of Engineering and Applied Technologies, Higher School of Technology, M′ghila Campus, Sultan Moulay Slimane University, Beni Mellal, Morocco, universitesms.com; ^5^ OCP-Nutricrops, OCP Group, Casablanca, Morocco; ^6^ TEDAEEP Research Team, Department of Life Sciences, Polydisciplinary Faculty of Larache, Abdelmalek Essaadi University, Larache, Morocco, uae.ma

**Keywords:** antioxidant activity, Beni Mellal—Khenifra, carob ripening, *Ceratonia siliqua* L., fruits′ trait, harvest timing

## Abstract

Carob fruit (*Ceratonia siliqua* L.) undergoes numerous physicochemical and biochemical transformations during its ripening; however, these changes have not previously been characterized in a temporal sequence that describes the states of maturity of carob fruit, limiting the potential to determine periods of harvest that can maximize both nutrient‐dense value and yield. In the current study, physical, colorimetric, and biochemical measurements were conducted that assessed the changes that occurred in unripe carob pods over five different carob pod ages (T1–T5). Physical traits including pod length, width, thickness, and mass all increased consistently throughout immature stages, reaching their maximum at T5. Brix and pH remained consistent until the early stages of maturity but began to decline beginning at T4, with Brix being the most impacted of the measurements. Total sugars varied across stages, with a minimum of 1616 mg measured in T2 and a maximum of 2198 mg measured in T3. The biochemical results showed that total phenolics, flavonoids, condensed tannins, and even their antioxidant activity were all highest at T2, declining at all other maturity periods. These findings indicate that immature carob fruits experience various developmental phases, with the T2–T3 gap recognized as a critical period marked by heightened amounts of bioactive chemicals. This crucial period presents advantageous prospects for focused enhancement in food and pharmaceutical sectors. This study addresses a significant gap in the literature by clarifying the dynamic changes throughout the immature stage, so establishing a scientific foundation for the efficient utilization of immature carob pods and ensuring their sustained functional and agronomic value.

## 1. Introduction

The carob tree (*Ceratonia siliqua* L.), an evergreen species of the subfamily Caesalpinioideae in the Leguminosae family [1], is native to the western regions of Asia, its original habitat, and has since naturally expanded throughout the Mediterranean basin [2]. Renowned for its resilience to environmental stressors, including drought tolerance and the ability to thrive in poor soils′ fertility, carob is increasingly valued for its substantial ecological, nutritional, and medicinal significance [3]. Moreover, owing to its deep‐rooting system, evergreen canopy, and previous distinctive agroecological adaptations, this species plays a pivotal role in mitigating soil erosion, land degradation, and desertification in vulnerable ecosystems [4–6]. Moreover, carob fruit has garnered increasing attention in food science and technology, as reflected by a surge of recent studies exploring its composition, functional properties, and value‐added derivatives.

The carob pod, consisting on average of 90% pulp and 10% seeds, encases its seed within the pericarp and mesocarp and is distinguished by its numerous applications [7]. The chemical composition of carob pulp varies considerably with cultivar, climatic conditions, and geographic origin. Both soil type and the degree of fruit ripeness at harvest influence the nutritional composition and the bioactive compounds in the pulp, ultimately shaping its biochemical profile. These variations reflect the combined effects of genetic and environmental factors [8–10]. Carob pulp and seeds are distinguished by their high polyphenol content, which confers a range of health‐promoting effects [11], including improved digestion [12], and reduced blood cholesterol [13] and glucose levels [14]. With a high pulp sugar content (48%–56%) [15], carob serves as a valuable ingredient in the culinary sector, commonly used in syrups and as a natural alternative to chocolate in cookies and ice cream formulations [16–18]. Carob flour is employed in the production of dietetic and gluten‐free products. A substantial body of research has demonstrated the utilization of carob fruit and its by‐products as a functional food and food additive [1]. However, its substantial dietary fiber content (27%–50%) [15], further imparts health‐promoting properties, supporting glycemic control, lipid regulation, and cancer prevention and underscoring its potential for pharmaceutical applications [19]. Carob seeds are rich in galactomannan (80%–85%) [20], a polysaccharide commercially known as E410 and widely employed in food technology as a stabilizer, thickener, and gelling agent. Beyond the food industry, galactomannan also finds broad applications in cosmetics, textiles, pharmaceuticals, and paint manufacturing, underscoring its industrial versatility [21]. Carob fruit is also a rich source of natural antioxidants, offering effective defense against reactive oxygen species, including both free and non‐free radicals.

Previous studies have consistently reported that immature carob fruits contain significantly higher levels of bioactive compounds, including phenolic acids, flavonoids, and tannins, than fully mature fruits [22–24]. Notably, Rtibi et al. [23] demonstrated that aqueous extracts of immature carob pulp exhibit antidiabetic properties by inhibiting intestinal glucose absorption and lowering blood glucose levels. The elevated antioxidant content in immature pods also suggests potential anticancer, antibacterial, and broader therapeutic applications, reinforcing their growing relevance in pharmaceutical and agro‐food industries [25]. Immature pods contain lower sugar due to active growth, whereas ripe pods accumulate sucrose, enhancing carbohydrate content and yield potential [26]. These contrasting profiles highlight the dynamic changes in physical and biochemical traits during maturation and underscore the need to optimize harvest timing. Although stage‐specific characterization guides optimal harvest decisions, the temporal dynamics of these transitions remain insufficiently understood.

Building on previous findings, this study investigates the temporal dynamics of carob (*C*. *siliqua* L.) fruit maturation and their influence on pomological traits, nutraceutical potential, and biochemical composition. By examining changes in key bioactive constituents, including total sugars, total polyphenols, total flavonoids, and condensed tannins, this study is aimed at characterizing the antioxidant capacity of immature carob pods from cultivars grown in the Beni Mellal—Khenifra region of Morocco. The overarching objective is to identify stage‐specific compositional profiles that support the valorization of unripe carob pod across diverse sectors, including the food, pharmaceutical, and functional ingredient industries.

## 2. Materials and Methods

### 2.1. Study Area, Plant Material and Sampling

Wild unripe carob pods were collected from three areas in the Ouaouizerth commune (32°11 ^′^52.9 ^″^N 6°19 ^′^52.2 ^″^W), located in the Béni‐Mellal—Khénifra region in the center of Morocco. The sampling period, conducted from April to June 2023, was designed to capture the full progression of carob pod maturation across distinct stages. Sampling was performed at approximately 15‐day intervals, depending on variations in the targeted physicochemical and biochemical parameters. The sampling dates were as follows (MM/DD): T1: 04/01–04/15, T2: 04/15–04/30, T3: 05/01–05/15, T4: 05/15–05/30, and T5: 06/01–06/15.

**Figure 1:**
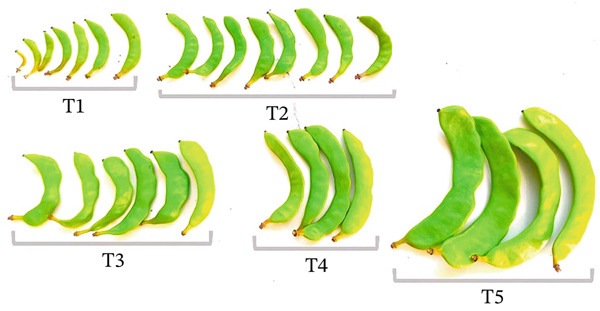
Maturation stages (T1–T5) of unripe carob pods examined in this study.

The development of immature carob pods was classified into five stages (T1–T5) based on a combination of pomological and colorimetric traits, monitored throughout the immature phase. This stage‐based framework provides a reproducible and physiologically meaningful classification in the absence of previously established standards for immature carob pods (Figure 1):

- T1 corresponds to the onset of active pod growth, immediately following the end of dormancy, marked by resumption of metabolic activity and rapid tissue expansion. Fruits at this stage display a bright green coloration.
- T2–T4 represents successive immature developmental phases, characterized by progressive increases in pod size and weight, with gradual changes in pulp color from bright green.
- T5 is defined as the final stage of the immature phase, representing a biologically significant transition toward ripening. It is characterized as the stage immediately preceding external browning; at this point, the fruit has not yet entered the post‐immature phase and exhibits a predominantly green color.

To ensure representative sampling, 10 trees were randomly selected within each of the three study areas. At each developmental stage, 50 carob pods were systematically collected per tree. Furthermore, samples were evenly collected from the four cardinal orientations (north, south, east, and west) and across the vertical profile of the canopy to minimize sampling bias and ensure representativeness.

A rigorous selection protocol was implemented to ensure that only healthy, disease‐free pods were included, thereby minimizing variability unrelated to developmental stage. Sampling was conducted by a team of botanists and taxonomists, and a voucher specimen was preserved and deposited in a recognized herbarium.

### 2.2. Plant Morphological Trait Measurement

Pomological analyses were conducted on a randomly selected subset of 15 carob pods per developmental stage, organized into three biological replicates of five pods each. These measurements were performed to characterize the morphological evolution of the fruit during maturation. The evaluated traits included pod mass, length, width, and thickness. In addition, seed yield (%) was calculated as the ratio of seed mass to total pod mass.

### 2.3. Physicochemical and Biochemical Analyses

#### 2.3.1. Color Attributes

The external color of unripe carob pods was assessed using the CIELAB color space (L ^∗^ “lightness”, a ^∗^ “green‐red”, b ^∗^ “blue‐yellow”) with a Minolta Chroma Meter CR‐400 (Minolta Corp., Osaka, Japan), a standard instrument for precise colorimetric analysis [27]. Color measurements were taken from three predefined surface areas on each pod to capture spatial variation and to monitor pigment changes throughout maturation. From the primary CIELAB coordinates, chroma (C ^∗^) and hue angle (h°); indicators of color intensity and tone, were subsequently calculated using standard equations (Equations 1 and 2), providing quantitative insights into the visual ripening progression:

(1) C∗=a∗2+b∗212/

(2) h°=tan−1ba

#### 2.3.2. pH and Total Soluble Solids (TSS)

Fruits from each developmental stage were sampled for physicochemical analysis, specifically pH and TSS. Juice was extracted using a Mellerware juice extractor (Mellerware, South Africa) to ensure uniform sample preparation. The pH was measured at room temperature using a calibrated pH meter (Thermo Orion 3 Star, United States), providing insights into fruit acidity across maturation stages. TSS, expressed as °Brix, were measured using a digital refractometer (Mettler‐Toledo GmbH, Switzerland) and served as an indicator of sugar accumulation during fruit development [27].

#### 2.3.3. Total Sugar Content

The quantification of total sugars was conducted in accordance with the method established by Dubois (1956) [26], with minor adjustments. Following an initial dilemma, 0.5 mL of the juice extracted from the pod was mixed with 2.5 mL of sulfuric acid. Then, 0.5 mL of phenol (5% *w*/*v*) was added. Following the completion of the dark incubation, the reading was taken at 488 nm. This was accomplished by employing a Shimadzu‐UV‐2401 PC UV spectrophotometer.

#### 2.3.4. Total Phenolic Content (TPC)

For the experiment, a mixture consisting of 100 *μ*L of juice, 0.5 mL of Folin–Ciocalteu reagent and 0.4 mL of a sodium carbonate solution (7.5%) was subjected to incubation for a period of 20 min at ambient temperature. Following this, the mixture was measured for its ability to absorb light at a wavelength of 760 nm, using a Schimadzu‐UV‐2401 PC spectrophotometer. The amount of TPC was calculated by using a standard curve, and was expressed as mg gallic acid per 100 mL juice, using a slightly modified Folin–Ciocalteu colorimetric method [21].

#### 2.3.5. Total Flavonoid Content (TFC)

The flavonoid content was assessed using the AlCl_3_ procedure [28] with slight modifications. A volume of 0.5 mL of juice and 0.5 mL of AlCl_3_ (2% *w*/*v*) were mixed, shaken, and then incubated in the dark for a period of 5 min. Subsequently, the absorbances were measured at a wavelength of 430 nm using a Schimadzu‐UV‐2401 PC spectrophotometer. The flavonoid content was calculated and expressed as milligrams of catechin per 100 mL of juice.

#### 2.3.6. Total Condensed Tannins

To determine condensed tannins present in the carob juice, we adapted the method developed by Broadhurst and Jones [29]. We mixed 200 *μ*L juice with 1.5 mL of 4% methanol vanillin, and after that, we added 750 *μ*L of concentrated hydrochloric acid (37%). The mixture was kept in the dark for approximately 10 min, after which the absorbance was measured at 500 nm. The total amount of condensed tannins was measured and provided as catechin equivalents per 100 ml of carob juice.

### 2.4. Antioxidant Activity

The following three assays were employed: DPPH, ABTS, and FRAP were utilized to evaluate the antioxidant capacity of unripe carob pods.

#### 2.4.1. DPPH Assay

The DPPH assay was performed to determine the antioxidant capability of carob juice, conferring to the practice adopted by Szabo et al. [28]. A volume of 0.1 mL of juice, diluted 1000‐fold with 60% ethanol, was mixed with 2 mL of DPPH solution (0.1 mM). The mixture was then shaken and left to incubate for approximately 45 min. Absorbance was measured at a wavelength of 517 nm using a Schimadzu UV‐2410 PC spectrophotometer. A standard of Trolox (1–5 mg/mL) was utilized (*R* ^2^ = 0.9706), and the final values of free radical scavenging activity of juice samples were expressed as milligrams of Trolox equivalents (mg TE) per 100 mL of juice.

#### 2.4.2. ABTS Assay

We performed the ABTS assay according to the protocol of Uysal et al. [30] to determine the antioxidant capacity. First, we mixed a 7‐mM ABTS solution with 2.45‐mM potassium persulfate and then incubated it in the dark at normal room temperature for 12–16 h. Prior to the experiment, we diluted the solution with ethanol to absorb 0.700 ± 0.02 at 734 nm. To determine the ABTS scavenging activity as milligrams of Trolox equivalents per 100 milliliters of juice, 1 mL of extract from each juice sample was mixed with 2 mL of standard ABTS solution, and it was left to stay at ambient temperature for 30 min. The absorbance of the ABTS solution was measured at 734 nm. All measurements were performed in triplicate to ensure accuracy and reproducibility.

#### 2.4.3. FRAP Assay

Benzie and Strain′s [31] methodology was followed for doing a FRAP assay of carob juice samples to find out the antioxidant potential. First of all, 10‐mmol/L TPTZ, 40‐mmol/L FeCl_3_, and 0.3‐M acetate buffer (pH = 3.6) were combined in a 10:1:1 ratio to create the FRAP reagent. After combining the juice with 3 mL of recently made FRAP reagent, it was vortexed and allowed to incubate for 30 min. Trolox was used as a standard from 1 to 5 mg/mL (*R* ^2^ = 0.9706), and the absorbance was measured at 593 nm. The results were represented in mg TE/100 mL of juice.

### 2.5. Statistical Analysis

The data were obtained in triplicate and presented as means ± standard deviation. A one‐way analysis of variance (ANOVA) was performed using GraphPad Prism 8.0.1 software to assess significant differences between the groups. Pearson′s correlation analysis was conducted using R software (Version 4.4.3) (R Core Team, 2025) to evaluate the relationships among the measured traits, employing the correlation function from the stats package. To explore patterns of variation among sampling stations and maturity stages and to identify the variables most responsible for these differences, a principal component analysis (PCA) was performed using the FactoMineR package. Graphical outputs were generated with the factoextra package. Principal components (PCs) were retained for interpretation based on their eigenvalues (> 0.8), ensuring that only components explaining a substantial proportion of the total variance were considered.

## 3. Results and Discussion

### 3.1. Fruit Morphological/Physical Properties

The physical characteristics of carob fruit exhibited marked variation across maturation stages under Moroccan environmental conditions, reflecting progressive morphological changes during development (Table 1). Indeed, a significant difference in all analyzed traits was observed between the five ripening stages. At the final stage of unripe maturation (T5), carob pods reached maximum values across all measured physical parameters, including length (19.1 cm), width (1.88 cm), thickness (0.48 cm), and weight (9.92 g), alongside the highest seed yield, accounting for 13.20% of total pod mass. However, Stage T1 recorded the lowest values for pod length, width, and weight, with values of 13.01 cm, 1.48 cm, and 3.37 g, respectively. Similarly, pod thickness reached a minimum at Stage T2 (0.18 cm), whereas the lowest seed yield was observed at Stage T3, averaging just 2.7 g. During maturation, carob fruit exhibits a predictable morphological development, characterized by progressive increases in pod dimensions and associated yield components. This trend reflects the natural accumulation of biomass and resource allocation toward carob fruit and seed development during the later stages of ripening [9].

**Table 1:** Variation of carob fruit physical properties during maturation.

Trait	Stage				
	T1	T2	T3	T4	T5
Length (cm)	13.01 ± 2.7^c^	13.95 ± 0.84^c^	16.70 ± 2.64^b^	17.50 ± 1.02^ab^	19.10 ± 0.87^a^
Width (cm)	1.48 ± 0.32^b^	1.49 ± 0.12^b^	1.78 ± 0.22^a^	1.94 ± 0.06^a^	1.88 ± 0.07^a^
Thickness (cm)	0.20 ± 0.02^c^	0.18 ± 0.07^c^	0.23 ± 0.07^c^	0.38 ± 0.07^b^	0.48 ± 0.07^a^
Pod weight (g)	3.37 ± 1.43^d^	5.65 ± 1.03^c^	6.63 ± 0.89^bc^	7.33 ± 0.89^b^	9.92 ± 1.00^a^
Seed yield (%)	3.70 ± 0.2^d^	4.20 ± 0.8^c^	2.7 ± 0.3^c^	6.1 ± 0.6^b^	13.20 ± 0.1^a^

*Note:* Means of 15 unripe carob fruits in each row followed by different letters are significantly different (*p* < 0.05); ±, standard deviation.

Regarding the fruit color properties, there were significant differences in these traits during ripening (Table 2). Lightness (L ^∗^) recorded the lowest value during the T5 stage and the highest value during the T1 stage with averages of 72.48 and 77.12, respectively. Redness (a ^∗^, ± red–green) recorded the lowest value during the T2 stage and the highest value during the T5 stage with averages of −15.20 and −10.17, respectively. Yellow (b ^∗^, ± yellow‐blue) recorded the lowest value during stage T3 and the highest value during stage T5 with averages of 17.10 and 21.28, respectively. The color intensity (C ^∗^) recorded the lowest value during the T3 stage and the highest value during the T2 stage with averages of 20.37 and 30.29, respectively. However, the dominant color tone (H ^∗^) did not show any significant variability at any of the ripening stages analyzed. The unripe pods exhibit high lightness (L ^∗^) values at the early stages T1 and T2, followed by a significant decrease up to stage T5. This change in lightness at T5 reflects major biochemical reorganizations occurring during fruit maturation [9]. At this stage, the mechanical properties of the fruit, including pod and seed rigidity, have already reached advanced levels, although the external color has not yet begun to acquire its definitive dark hue.

**Table 2:** Variation of carob fruit color properties during maturation.

Trait	T1	T2	T3	T4	T5
L	77.12 ± 2.06^a^	74.10 ± 0.66^ab^	75.07 ± 0.75^ab^	74.47 ± 0.56^ab^	72.48 ± 0.76^b^
a	−11.47 ± 1.50^a^	−15.20 ± 0.20^b^	−11.07 ± 0.26^a^	−11.24 ± 0.17^a^	−10.17 ± 0.26^a^
b	18.66 ± 4.40^bc^	26.20 ± 0.82^a^	17.10 ± 0.72^c^	18.26 ± 0.42^bc^	21.28 ± 0.53^b^
C	21.92 ± 4.53^b^	30.29 ± 0.81^a^	20.37 ± 0.75^b^	21.44 ± 0.44^b^	23.58 ± 0.59^b^
H	−1.01 ± 0.05^a^	−1.05 ± 0.01^a^	−1.00 ± 0.01^a^	−1.02 ± 0.00^a^	−1.13 ± 0.00^a^

*Note:* Means of 15 unripe carob fruits in each row followed by different letters are significantly different (*p* < 0.05); ±, standard deviation.

Beyond Stage T5, during the postmaturation phase characterized by fruit desiccation, the continued darkening suggests the involvement of enzymatic oxidation of phenolic compounds, potentially catalyzed by enzymes such as polyphenol oxidase (PPO) and peroxidase (POX) [32].

### 3.2. Fruit Chemical and Biochemical Properties

Significant differences in the chemical and biochemical properties of carob fruits during ripening stages between April and June were reported in Tables 3 and 4. The lowest pH was observed during the T4 stage, whereas the highest value was recorded during the T1 and T5 stage with averages of 5.36 and 5.71, respectively. The lowest Brix° was observed during the T4 stage and the highest value was recorded during the T2 stage with averages of 6.08 and 8.41, respectively. The lowest total sugar content was observed during the T2 stage, whereas the highest value was recorded during the T3 stage with averages of 1616 mg and 2198 mg EG/100 mL of juice. Considering the overall trends in pH and Brix values across the five maturation stages, both parameters exhibit relative stability until Stage T4, where a marked decline is observed, particularly in Brix, and to a lesser extent in pH. This abrupt shift may reflect underlying biochemical transitions specific to this stage that were not directly assessed in the current study. Possible explanations include the onset of metabolic shifts such as the hydrolysis or redistribution of soluble sugars, increased respiration rates, or shifts in the balance of organic acids and polyphenolic compounds. Additionally, this phase may correspond to a transition in physiological sink‐source dynamics, where the fruit′s metabolic priority shifts from accumulation to mobilization or degradation. Although measurement error is unlikely given the use of pooled pods from multiple orientations and replicates, the lack of strong correlation between Brix, pH, and other studied traits suggests that unmeasured biochemical or enzymatic processes could be involved. Future studies integrating metabolomic profiling or enzymatic assays could provide further insights into the mechanisms driving this unexpected shift in compositional traits at the late maturation stage.

**Table 3:** Variation of carob fruit chemical properties during maturation.

	pH	Brix	TSC mg EG/100 mL
T1	5.718 ± 0.002^a^	8.08 ± 0.14^a^	1675 ± 116.3^b^
T2	5.624 ± 0.003^c^	8.41 ± 0.14^a^	1616 ± 67.74^b^
T3	5.634 ± 0.003^b^	8.08 ± 0.14^a^	2198 ± 16.76^a^
T4	5.362 ± 0.002^d^	6.08 ± 0.14^b^	2037 ± 91.65^a^
T5	5.707 ± 0.003^a^	8.18 ± 0.16^a^	2139 ± 38.53^a^

*Note:* Experiments were performed in triplicate (*n* = 3) and presented as mean ± SD. Conventional one‐way ANOVA with Tukey′s test at 5%. Means with different letters in the same row are significantly different (*p* < 0.05).

Abbreviation: TSC, total sugar content.

**Table 4:** Variation of carob fruit biochemical properties and antioxidant activity during maturation.

Stage	TPC (mg EAG/100 mL of juice)	TFC (mg EQ/100 mL of juice)	TCT (mg CE/100 mL of juice)	DPPH (mg TE/100 mL of juice)	ABTS (mg TE/100 mL of juice)	FRAP (mg TE/100 mL of juice)
T1	2594 ± 49.1^c^	784.4 ± 66.63^b^	536.5 ± 66.49^b^	6915 ± 570.7^b^	645.2 ± 50.63^ab^	140.4 ± 6.664^b^
T2	3573 ± 96.74^a^	1335 ± 187.9^a^	739.3 ± 81.5^a^	8469 ± 201.5^a^	703.2 ± 53.71^a^	240.0 ± 50.63^a^
T3	2893 ± 18.01^b^	924.1 ± 55.58^b^	611.8 ± 42.7^ab^	6600 ± 231.9^b^	553.6 ± 21.74^b^	97.35 ± 10.27^b^
T4	2719 ± 12.28^c^	244.4 ± 8.90^c^	708 ± 8.29^a^	6830 ± 153.7^b^	528.7 ± 73.95^b^	137.5 ± 4.475^b^
T5	2863 ± 31.74^b^	276.9 ± 7.69^c^	77.17 ± 8.6^c^	7201 ± 143.6^b^	700.6 ± 33.53^a^	145.9 ± 18.32^b^

*Note:* Data are presented as mean ± SD, based on triplicate experiments (*n* = 3). Distinct lowercase letters, in the same row, indicate statistically significant variations (*p* < 0.05) across provenances as determined by Tukey test.

Abbreviations: TCF, total flavonoid content; TCT, total condensed tannins; TE, Trolox equivalents; TPC, total phenolic content.

Our findings on the variation in total sugar content during carob fruit ripening are consistent with those reported by Vekiari et al. [32] for Turkish carobs and Othmen et al. [26] for Tunisian carobs, both of which highlight the strong influence of developmental stage on sugar accumulation. Ben Othmen et al. [26] demonstrated that sugar content increases progressively from the unripe stage (T1) to the ripe stage (T3), with sucrose concentrations peaking at T3 in two studied carob cultivars. In contrast, the lowest levels were observed at the T1 stage, averaging just 231 mg and 242.65 mg, respectively. As the fruit develops, the content of soluble sugars, particularly sucrose accumulation, increases progressively, playing a central role as a biochemical marker of carob fruit maturation and its potential utility in determining optimal harvest timing for sweetness and quality [33]. Conversely, previous studies have reported a decline in glucose and fructose concentrations during carob fruit maturation phases [23], a trend largely attributed to their involvement in polysaccharide biosynthesis and their integration into various metabolic pathways [34]. This shift in sugar profile is closely regulated by sucrose phosphate synthase (SPS) activity, which plays a central role in redirecting hexose sugars toward sucrose accumulation during the later stages of fruit development. Indeed, Gomez et al. [33] have demonstrated a synchronization between SPS activation and sucrose accumulation during ripening. During this phase, the fruit continuously receives sucrose produced by photosynthesis in the leaves, thus constituting the main biosynthetic pathway for this sugar. In addition, sucrose accumulation may also result from the breakdown of other carbohydrates stored in plant organs.

Total phenol content was lowest at Stage T1, whereas the highest value was recorded at stage T2 with mean values of 2594 mg and 3573 mg, respectively. TFC was lowest at stage T4, whereas the highest value was recorded at stage T2 with respective means of 244.40 mg and 1335 mg, respectively. Condensed tannin content was lowest at stage T5, whereas the highest value was recorded at Stage T2 with respective means of 77.17 mg and 739.30 mg, respectively. This characterization offers a valuable framework for identifying stage T2 as an optimal harvest point for maximizing specific bioactive traits, particularly total phenolics, flavonoids, and condensed tannins; compounds closely linked to the fruit’s antioxidant potential and functional value.

Consistent with our findings, Ben Othmen et al. [26] confirmed a pronounced decline in the total concentrations of polyphenols, flavonoids, and condensed tannins in carob pods as ripening progresses. The highest levels of these bioactive compounds were observed at the early unripe stage, averaging 5670 mg for total polyphenols, 2543.7 mg for flavonoids, and 1520 mg for condensed tannins, before gradually decreasing to their lowest concentrations at full maturity. Notably, the absolute content values measured in Moroccan carob pods in the present study were lower than those reported by Ben Othmen et al. [26], a discrepancy that may reflect a combination of genotypic variability, environmental influences such as altitude, temperature, and soil composition, and potential differences in extraction protocols or analytical sensitivity. These findings underscore the importance of local agroecological context in shaping the phytochemical composition of carob and suggest the need for further comparative studies to disentangle genetic and environmental contributions to trait variability. Supporting this trend, Kyriacou et al. [9] also reported that the highest concentrations of total polyphenols, flavonoids, and condensed tannins occurred at the unripe stage T1, with average values of 181 mg, 2551 mg, and 198 mg, respectively. Interestingly, these values are lower than those observed in the present study, further reinforcing the influence of regional, genetic, and methodological differences on the accumulation of bioactive compounds in carob pods.

The antioxidant capacity of green carob pod juice recorded the highest value using three tests DPPH, ABTS and FRAP during stage T2 with averages of 8469 mg, 703.2 mg and 240 mg, respectively. Although Stage T3 marked the lowest values for the DPPH and FRAP tests with averages of 6600 and 97.35 mg, respectively, the ABTS test was recorded during Stage T4 with an average of 528.7 mg. In line with this study′s results, Ben Othmen et al. [35] reported that antioxidant activity in carob pods is strongly influenced by the stage of ripening. The highest levels of free radical scavenging activity were recorded at the early maturity stages, reaching up to 3278 mg, whereas a sharp decline was observed as ripening advanced, with values dropping to between 11 and 4.56 mg at full maturity. These observations are further corroborated by the work of Benchikh et al. [20], who reported similar declines in antioxidant potential across ripening stages in carob pods. The observed trend closely reflects the progressive depletion of bioactive compounds, most notably polyphenols, partly driven by the activation of PPO, an enzyme that catalyzes the oxidation of phenolic substrates [34]. This enzymatic activity underscores the strong association between antioxidant capacity and phenolic content throughout fruit maturation. At the start of ripening, these compounds exhibit relatively low polarity; however, as maturation advances, their progressive condensation increases polarity, a transformation consistent with their involvement in enzymatic browning reactions. These results suggest that carob pulp can serve as a valuable natural resource for the prevention or mitigation of oxidative stress‐related disorders [36]. Importantly, the pronounced antioxidant potential observed at the early stages underscores the considerable nutritional and functional value of unripe carob pods prior to maturity, providing key insights for both nutritional applications and the development of functional foods. Overall, these findings emphasize the stage‐dependent functional properties of carob fruits, which could be strategically exploited in health‐promoting dietary formulations and industrial applications.

### 3.3. Trait Interrelationships During Carob Fruit Maturation: Toward an Optimized Multitrait Harvest Strategy

**Figure 2:**
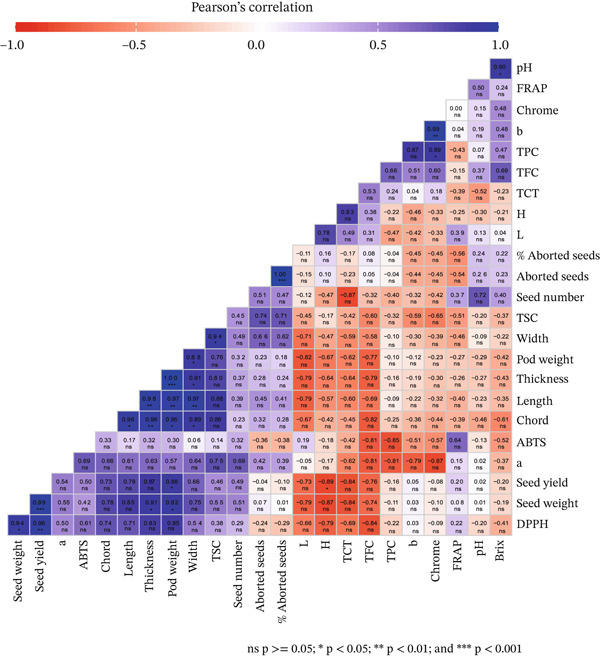
Correlation coefficients between the variation of the physical, biochemical, and antioxidant traits during fruit maturation stages.

Multivariate analyses were conducted to explore the relationships between the physical, biochemical, antioxidant and color properties of Moroccan carob fruit at different maturation stages. Pearson′s correlation analysis was employed to assess the degree of association between key traits (Figure 2). This approach provided insights into how biochemical changes during maturation are interconnected and how these traits collectively influence fruit quality. The results revealed a significant association among the physical, biochemical, and antioxidant traits of carob fruit during maturation. Strong positive correlations were observed among pod weight, width, length and thickness (*r* > 0.9, *p* < 0.001), indicating that these traits increase simultaneously as the fruit develops. Similarly, seed weight and seed yield were highly correlated (*r* = 0.99, *p* < 0.001), suggesting that higher seed production directly contributes to greater seed biomass. In contrast, the percentage of aborted seeds was negatively correlated with seed weight and yield (*r* = −0.7, *p* < 0.001), implying that increased seed abortion reduces overall seed output. Biochemical composition displayed distinct trends, with TSS (TSC) positively correlating with pod length and width with a coefficient of 0.94 (*p* < 0.001), indicating a possible synergy between sugar accumulation and fruit development. TPC and TFC were positively correlated (*r* ≈0.7), suggesting that flavonoids contribute significantly to the total polyphenol pool. Furthermore, the fruit antioxidant activities (DPPH) during maturation stages were positively associated with the seed yield (*r* > 0.9, *p* < 0.001). Variation in color parameters showed strong positive correlations with TPC, indicating that immature fruits contain higher phenolic concentrations. These findings offer valuable insights into the biochemical and physical changes that occur during carob fruit maturation and highlight key quality markers relevant for fruit processing and nutritional applications.

### 3.4. Carob Fruit Trait Integration and Divergence During Maturation Stages: A Multivariate Perspective

**Figure 3:**
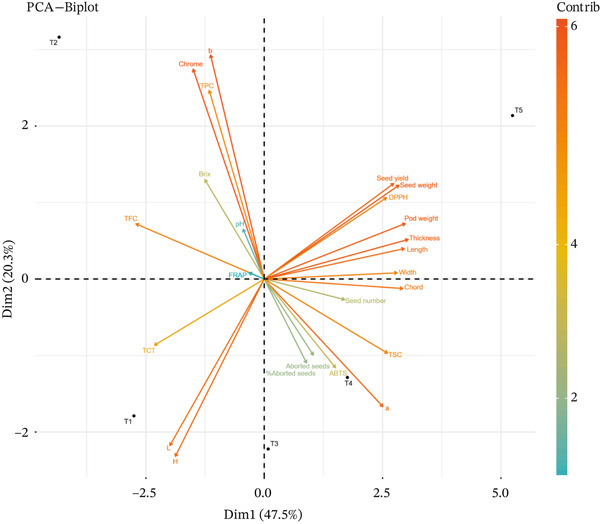
Principal component analysis map of physicochemical and antioxidant traits in carob fruit during maturation.

The PCA (Table 5 and Figure 3) revealed four PCs that collectively explain 100% of the variance in the dataset, highlighting the key factors influencing carob fruit maturation. The PC loadings of more than |0.8| were considered as being significant for each variable. The first component (PC1) explains more than 47.47% of the total variability and it is predominantly associated with fruit physical traits, including length (0.941), width (0.893), thickness (0.967), pod weight (0.947), and seed weight (0.905), as well as seed yield (0.870), suggesting that this component reflects fruit and seed size, biomass accumulation, and pod development. The second component (PC2) explains about 20.30% of the total variability and it is mainly correlated with color parameters (b: 0.931, chroma: 0.872) and TPC (TPC: 0.784), indicating its role in fruit pigmentation and biochemical composition. The third component (PC3) explains more than 17.29% of the total variability and it is associated with seed abortion traits (% aborted seeds: 0.824, aborted seeds: 0.811) and antioxidant activity (FRAP: −0.848, ABTS: −0.796), reflecting seed viability and oxidative stress response mechanisms. The last component (PC4) presents about 14.94% of the total variability and it is strongly linked to pH (0.968) and soluble solids (°Brix: 0.777), suggesting that it captures fruit acidity and sugar accumulation dynamics. Notably, the first two PCs explain 67.77% of the total variance, emphasizing the dominant influence of fruit size, seed weight, and color traits during fruit maturation. Additionally, the inverse relationship observed between TFC (TFC; −0.862 in PC1) and TPC (TPC; 0.784 in PC2) suggests a potential trade‐off between flavonoid accumulation and other biochemical traits. The distinct clustering of seed‐related parameters in PC3 and the sugar–acidity balance in PC4 further indicates that seed development and fruit sweetness evolve independently of morphological and color variations. Collectively, these findings provide valuable insights into the physiological, biochemical, and morphological drivers of fruit quality and yield during maturation, enhancing our understanding of carob fruit development.

**Table 5:** Eigenvectors of principal components from PCA analysis based on the analyzed traits during fruit maturation.

Parameter	PC1	PC2	PC3	PC4
Length	**0.941**	0.126	0.284	−0.131
Width	**0.893**	0.026	0.449	0.014
Thickness	**0.967**	0.164	0.095	−0.170
Chord	**0.931**	−0.039	0.147	−0.331
Pod weight	**0.947**	0.231	0.078	−0.209
Seed number	0.540	−0.086	0.004	**0.837**
Aborted seeds	0.330	−0.316	**0.811**	0.365
% Aborted seeds	0.284	−0.349	**0.824**	0.344
Seed weight	**0.905**	0.390	−0.160	0.061
Seed yield	**0.870**	0.397	−0.285	0.068
L	−0.634	−0.692	−0.286	0.193
a	**0.797**	−0.530	−0.132	0.257
b	−0.361	**0.931**	−0.009	−0.055
Chrome	−0.478	**0.872**	0.025	−0.103
H	−0.597	−**0.737**	0.196	−0.248
PH	−0.142	0.207	0.028	**0.968**
Brix	−0.399	0.413	0.257	**0.777**
TPC	−0.369	**0.784**	0.473	−0.160
TFC	**−0.862**	0.229	0.409	0.194
TCT	**−0.737**	−0.275	0.181	−0.590
TSC	**0.828**	−0.308	0.466	−0.037
DPPH	**0.822**	0.338	**−**0.435	−0.144
ABTS	0.479	−0.369	**−0.796**	0.039
FRAP	−0.102	0.026	**−0.848**	0.520
Eigenvalue	11.393	4.871	4.150	3.586
Variance %	47.472	20.295	17.293	14.941
Cumulative variance %	47.472	67.767	85.059	100.000

*Note:* Significant eigenvectors, higher than |0.8|, are marked in bold.

## 4. Conclusion

A complete description of the physical, biochemical, and functional changes that accompany the maturation of unripe carob fruits has been accomplished. Fruit weight and size continue to increase until fruits are fully mature, whereas parameters such as pH, °Brix, and soluble sugars increase until early to midmaturation.

Bioactive compounds (total phenols, flavonoids, and condensed tannins) are accumulated in the fruit prior to ripening but are depleted during the ripening process, probably via metabolically driven enzymatic oxidation, with antioxidant activity also being maximally present during early stages of fruit development. By integrating agronomic, physiological, and biochemical parameters, candidate harvesting windows for unripe carob fruits have been determined. Intermediate ripening stages (T2–T3) maintain high levels of bioactive compounds, sugars, and °Brix, thereby enhancing the nutritional value and functional properties that carob fruits can provide to consumers.

Ripening stages (T3–T5) promote the accumulation of sugars and yield‐related components, highlighting trade‐offs that may be of interest to both industry and agronomists. Thus, precision harvesting windows have been determined for unripe carob fruits that target the use of the fruits in foods, functional foods or pharmacological applications, while also providing indications of how best to optimize value chains for the processing and processing of these fruits.

## Author Contributions

Conceptualization: S.L., K.E., C.E‐R., and A.A.; methodology: A.A., S.L., and A.H.; software: M.L., C.E‐R., A.M., and W.S.; validation: A.E‐D., S.S., K.E., and A.A.; formal analysis: C.J., W.S., and A.M.; investigation: C.J., M.L., C.E‐R., and M.K.; resources: M.K., and C.E‐R.; data curation: A.M. and A.H.; writing—original draft preparation: S.L., A.A., C.E‐R., and A.H.; writing—review and editing: S.L., S.S., K.E., A.M, and A.E‐D.; visualization: S.S.; supervision: S.S. and K.E.; project administration: A.M., S.S., and K.E.; funding acquisition: K.E. S.L. and A.A. have contributed equally to this work.

## Funding

This study was supported by the National Institute of Agricultural Research of Morocco (INRA).

## Disclosure

All authors have read and agreed to the published version of the manuscript.

## Conflicts of Interest

The authors declare no conflicts of interest.

## Data Availability

All data generated or analyzed during this study are included in this published article.

## References

[1] Basharat Z. et al., Nutritional and Functional Profile of Carob Bean (Ceratonia siliqua): A Comprehensive Review, International Journal of Food Properties. (2023) 26, no. 1, 389–413, 10.1080/10942912.2022.2164590.

[2] Fadel A. H. I. , Kamarudin M. S. , Romano N. , Ebrahimi M. , Saad C. R. , and Samsudin A. A. , Carob Seed Germ Meal as a Partial Soybean Meal Replacement in the Diets of Red Hybrid Tilapia, Egyptian Journal of Aquatic Research. (2017) 43, no. 4, 337–343, 10.1016/J.EJAR.2017.09.007.

[3] Gioxari A. , Carob: A Sustainable Opportunity for Metabolic Health, Foods. (2022) 11, no. 14, 1–17, 10.3390/foods11142154. PMC9325207 35885396

[4] Battle J. I. and Tous , Carob Tree. Ceratonia siliqua L. Promoting the Conservation and Use of Underutilized and Neglected Crops, 1997, International Plant Genetic Resources Institute, 10.1007/978-0-85729-323-7_1829.

[5] Owen R. W. , Haubner R. , Hull W. E. , Erben G. , Spiegelhalder B. , Bartsch H. , and Haber B. , Isolation and Structure Elucidation of the Major Individual Polyphenols in Carob Fibre, Food and Chemical Toxicology. (2003) 41, no. 12, 1727–1738, 10.1016/S0278-6915(03)00200-X, 14563398. 14563398

[6] El Kah R. , Profile S. , Rachid Z. , and Diouri M. , Morphological and Biochemical Characterization of Morocco Carob Tree (Ceratonia siliqua L.), International Journal of Biological and Medical Research. (2015) 6, no. 2, 4946–4952, https://www.researchgate.net/publication/277775017.

[7] Laaraj S. , Hussain A. , Mouhaddach A. , Noutfia Y. , Gorsi F. I. , Yaqub S. , Hussain I. , Nisar R. , Salmaoui S. , and Elfazazi K. , Nutritional Benefits and Antihyperglycemic Potential of Carob Fruit (*Ceratonia siliqua* L.) - An Overview, Ecological Engineering and Environmental Technology. (2024) 25, no. 3, 124–132, 10.12912/27197050/178456.

[8] Correia P. J. , Saavedra T. , Gama F. , da Graça Miguel M. , de Varennes A. , and Pestana M. , Biologically Active Compounds Available in Ceratonia siliqua L. Grown in Contrasting Soils Under Mediterranean Climate, Scientia Horticulturae. (2018) 235, 228–234, 10.1016/J.SCIENTA.2018.03.010.

[9] Kyriacou M. C. , Antoniou C. , Rouphael Y. , Graziani G. , and Kyratzis A. , Mapping the Primary and Secondary Metabolomes of Carob (Ceratonia siliqua L.) Fruit and Its Postharvest Antioxidant Potential at Critical Stages of Ripening, Antioxidants. (2021) 10, no. 1, 10.3390/antiox10010057, 33466561. PMC7824902 33466561

[10] Ortega N. , Macià A. , Romero M. P. , Trullols E. , Morello J. R. , Anglès N. , and Motilva M. J. , Rapid Determination of Phenolic Compounds and Alkaloids of Carob Flour by Improved Liquid Chromatography Tandem Mass Spectrometry, Journal of Agricultural and Food Chemistry,. (2009) 57, no. 16, 7239–7244, 10.1021/jf901635s, 19624131. 19624131

[11] Slavin J. , Fiber and Prebiotics: Mechanisms and Health Benefits, Nutrients. (2013) 5, no. 4, 1417–1435, 10.3390/nu5041417, 23609775. 23609775 PMC3705355

[12] Raninen K. , Lappi J. , Mykkänen H. , and Poutanen K. , Dietary Fiber Type Reflects Physiological Functionality: Comparison of Grain Fiber, Inulin, and Polydextrose, Nutrition Reviews. (2011) 69, no. 1, 9–21, 10.1111/J.1753-4887.2010.00358.X, 21198631. 21198631

[13] de Bock M. , Derraik J. G. B. , and Cutfield W. S. , Polyphenols and Glucose Homeostasis in Humans, Journal of the Academy of Nutrition and Dietetics. (2012) 112, no. 6, 808–815, 10.1016/J.JAND.2012.01.018. 22709808

[14] Kaderi M. , Ben Hamouda G. , Zaeir H. , Hanana M. , and Hamrouni L. , Notes ethnobotanique et phytopharmacologique sur Ceratonia siliqua (L.), Phytotherapie. (2015) 13, no. 2, 144–147, 10.1007/s10298-014-0904-4.

[15] Bengoechea C. , Romero A. , Villanueva A. , Moreno G. , Alaiz M. , Millán F. , Guerrero A. , and Puppo M. C. , Composition and Structure of Carob (Ceratonia siliqua L.) Germ Proteins, Food Chemistry. (2008) 107, no. 2, 675–683, 10.1016/j.foodchem.2007.08.069.

[16] Elfazazi K. , Harrak H. , Achchoub M. , and Benbati M. , Physicochemical Criteria, Bioactive Compounds and Sensory Quality of Moroccan Traditional Carob Drink, Materials Today: Proceedings. (2020) 27, 3249–3253, 10.1016/j.matpr.2020.04.868.

[17] Goulas V. , Stylos E. , Chatziathanasiadou M. V. , Mavromoustakos T. , and Tzakos A. G. , Molecular Sciences Functional Components of Carob Fruit: Linking the Chemical and Biological Space, International Journal of Molecular Sciences. (2016) 17, no. 11, 10.3390/ijms17111875, 27834921. PMC5133875 27834921

[18] Maier H. , Anderson M. , Karl C. , Magnuson K. , and Whistler R. L. , Guar, Locust Bean, Tara, and Fenugreek Gums, Industrial gums, 2013, Academic Press, 181–226, 10.1016/B978-0-08-092654-4.50012-7.

[19] Laaraj S. , Influence of Harvesting Stage on Phytochemical Composition, Antioxidant, and Antidiabetic Activity of Immature Ceratonia siliqua L. Pulp From Béni Mellal-Khénifra Region, Morocco: In Silico, In Vitro, and In Vivo Approaches, Current Issues in Molecular Biology. (2024) 46, no. 10, 10991–11020, 10.3390/cimb46100653, 39451533. 39451533 PMC11506481

[20] Benchikh Y. , Louaileche H. , George B. , and Merlin A. , Changes in Bioactive Phytochemical Content and In Vitro Antioxidant Activity of Carob (Ceratonia siliqua L.) as Influenced by Fruit Ripening, Industrial Crops and Products. (2014) 60, 298–303, 10.1016/j.indcrop.2014.05.048.

[21] Farag M. A. , el-Kersh D. M. , Ehrlich A. , Choucry M. A. , el-Seedi H. , Frolov A. , and Wessjohann L. A. , Variation in Ceratonia siliqua Pod Metabolome in Context of Its Different Geographical Origin, Ripening Stage and Roasting Process, Food Chemistry. (2019) 283, 675–687, 10.1016/j.foodchem.2018.12.118, 30722926. 30722926

[22] Ydjedd S. , Bouriche S. , López-Nicolás R. , Sánchez-Moya T. , Frontela-Saseta C. , Ros-Berruezo G. , Rezgui F. , Louaileche H. , and Kati D. E. , Effect of In Vitro Gastrointestinal Digestion on Encapsulated and Nonencapsulated Phenolic Compounds of Carob (Ceratonia siliqua L.) Pulp Extracts and Their Antioxidant Capacity, Journal of Agricultural and Food Chemistry. (2017) 65, no. 4, 827–835, 10.1021/acs.jafc.6b05103, 28094929. 28094929

[23] Rtibi K. , Selmi S. , Grami D. , Saidani K. , Sebai H. , Amri M. , Eto B. , and Marzouki L. , Ceratonia siliqua L. (Immature Carob Bean) Inhibits Intestinal Glucose Absorption, Improves Glucose Tolerance and Protects Against Alloxan-Induced Diabetes in Rat, Journal of the Science of Food and Agriculture. (2017) 97, no. 8, 2664–2670, 10.1002/jsfa.8091, 27739095. 27739095

[24] Ikram A. , Khalid W. , Wajeeha Zafar K. U. , Ali A. , Afzal M. F. , Aziz A. , Faiz ul Rasool I. , al-Farga A. , Aqlan F. , and Koraqi H. , Nutritional, Biochemical, and Clinical Applications of Carob: A Review, Food Science and Nutrition. (2023) 11, no. 7, 3641–3654, 10.1002/FSN3.3367, 37457186. 37457186 PMC10345664

[25] El-Rhouttais C. , Elfazazi K. , Kettabi Z. E. , Laaraj S. , Elgoulli M. , Al-Zharani M. , Nasr F. A. , Qurtam A. A. , Bouslihim Y. , and Salmaoui S. , Effect of Xanthan Gum-Based Edible Coating Enriched With Cloves and Cinnamon for Extending the Shelf-Life of Pomegranate Fruit During Cold Storage, Scientific Reports. (2025) 15, no. 1, 31518, 10.1038/s41598-025-15467-x, 40858726. 40858726 PMC12381057

[26] Dubois M. , Gilles K. A. , Hamilton J. K. , Rebers P. A. , and Smith F. , Colorimetric Method for Determination of Sugars and Related Substances, Analytical Chemistry. (1956) 28, no. 3, 350–356, 10.1021/ac60111a017.

[27] Tikent A. , Antioxidant Potential, Antimicrobial Activity, Polyphenol Profile Analysis, and Cytotoxicity Against Breast Cancer Cell Lines of Hydro-Ethanolic Extracts of Leaves of (Ficus carica L.) From Eastern Morocco, Frontiers in Chemistry. (2024) 12, 10.3389/fchem.2024.1505473, 39665002. PMC11631630 39665002

[28] Szabo K. , Diaconeasa Z. , Cătoi A. F. , and Vodnar D. C. , Screening of Ten Tomato Varieties Processing Waste for Bioactive Components and Their Related Antioxidant and Antimicrobial Activities, Antioxidants. (2019) 8, no. 8, 10.3390/antiox8080292. PMC6719044 31398838

[29] Broadhurst R. B. and Jones W. T. , Analysis of Condensed Tannins Using Acidified Vanillin, Journal of the Science of Food and Agriculture. (1978) 29, no. 9, 788–794, 10.1002/jsfa.2740290908.

[30] Uysal S. , Zengin G. , Aktumsek A. , and Karatas S. , Chemical and Biological Approaches on Nine Fruit Tree Leaves Collected From the Mediterranean Region of Turkey, Journal of Functional Foods. (2016) 22, 518–532, 10.1016/j.jff.2016.02.006.

[31] Benzie I. F. F. and Strain J. J. , The Ferric Reducing Ability of Plasma (FRAP) as a Measure of ‘Antioxidant Power’: The FRAP Assay, Analytical Biochemistry. (1996) 239, no. 1, 70–76, 10.1006/abio.1996.0292. 8660627

[32] Vekiari S. A. , Ouzounidou G. , Ozturk M. , and Görk G. , Variation of Quality Characteristics in Greek and Turkish Carob Pods During Fruit Development, Procedia-Social and Behavioral Sciences. (2011) 19, 750–755, 10.1016/j.sbspro.2011.05.194.

[33] Gomez M. , Lajolo F. , and Cordenunsi B. , Evolution of Soluble Sugars During Ripening of Papaya Fruit and Its Relation to Sweet Taste, Journal of Food Science. (2002) 67, no. 1, 442–447, 10.1111/J.1365-2621.2002.TB11426.X.

[34] Gull J. , Sultana B. , Anwar F. , Naseer R. , Ashraf M. , and Ashrafuzzaman M. , Variation in Antioxidant Attributes at Three Ripening Stages of Guava (Psidium guajava L.) Fruit From Different Geographical Regions of Pakistan, Molecules. (2012) 17, no. 3, 3165–3180, 10.3390/MOLECULES17033165, 22418924. 22418924 PMC6268954

[35] Ben Othmen K. , Elfalleh W. , Lachiheb B. , and Haddad M. , Evolution of Phytochemical and Antioxidant Activity of Tunisian Carob (Ceratonia siliquaL.) Pods During Maturation, Eurobiotech Journal. (2019) 3, no. 3, 135–142, 10.2478/ebtj-2019-0016.

[36] Laaraj S. , Tikent A. , and El-rhouttais C. , Nutritional Value, HPLC-DAD Analysis and Biological Activities of Ceratonia siliqua L. Pulp Based on In Vitro and In Silico Studies, Scientific Reports. (2024) 14, no. 1, 31115, 10.1038/s41598-024-82318-6, 39732748. 39732748 PMC11682268

